# Malay sentiment analysis based on combined classification approaches and Senti-lexicon algorithm

**DOI:** 10.1371/journal.pone.0194852

**Published:** 2018-04-23

**Authors:** Ahmed Al-Saffar, Suryanti Awang, Hai Tao, Nazlia Omar, Wafaa Al-Saiagh, Mohammed Al-bared

**Affiliations:** 1 Faculty of Computer System and Software Engineering, University Malaysia Pahang UMP, Pahang, Malaysia; 2 Faculty of Information Science and Technology, Universiti Kebangsaan Malaysia UKM, Bangi, Selangor, Malaysia; Nanyang Technological University, SINGAPORE

## Abstract

Sentiment analysis techniques are increasingly exploited to categorize the opinion text to one or more predefined sentiment classes for the creation and automated maintenance of review-aggregation websites. In this paper, a Malay sentiment analysis classification model is proposed to improve classification performances based on the semantic orientation and machine learning approaches. First, a total of 2,478 Malay sentiment-lexicon phrases and words are assigned with a synonym and stored with the help of more than one Malay native speaker, and the polarity is manually allotted with a score. In addition, the supervised machine learning approaches and lexicon knowledge method are combined for Malay sentiment classification with evaluating thirteen features. Finally, three individual classifiers and a combined classifier are used to evaluate the classification accuracy. In experimental results, a wide-range of comparative experiments is conducted on a Malay Reviews Corpus (MRC), and it demonstrates that the feature extraction improves the performance of Malay sentiment analysis based on the combined classification. However, the results depend on three factors, the features, the number of features and the classification approach.

## Introduction

Malay language is an official statute in Malaysia and it widely used also in Brunei, Indonesia, and Singapore. The number of Malaysian internet users has a huge growth been in, 2006 the number users were 5 million to become more than 20 million users in the Dec.31, 2014 in Malaysia (http://www.internetworldstats.com in 2016). This growth of the Malay language in internet content has opened opportunities and interest for Malayan businesses to have a more feedback on their products from the customers that need more study on improving the Malay sentiment analysis technology. Malay sentiment analysis is also a key component of recommender systems for books, movies or music. Eventually, this approach leads to an increase in income for businesses and better quality of judgment for consumers. Developing robust models that capable of analyzing sentiment in real-time is one of the most dynamic research fields these days[1].

Sentiment Analysis (SA) carried out by studying the prevalence of positive or negative sentiments in expressing attitudes and opinions [2, 3]. There are two approaches for (SA) that automatically classify the text into positive or negative emotions: the semantic orientation (SO) and the machine learning (ML) approaches [4, 5]. Nevertheless, an integration between SO and ML obtained accurate results in the literature [6–11] due to the improving process of extracting features. Another superior approach to enhance the performance of SA is a combination of more than one traditional classifier. This combined technique such as voting and stacking was reported to achieve higher accuracy that outperforms other individual techniques in SA task [12–14]. The problem of the sentiment analysis in classification phase along with feature extraction or selection phase led us to investigate the effect of classifiers with the features. For some classifier such as K-NN [15], researchers obtained acceptable results. In addition, there is a need to explore a combination of several classifiers to obtain better result. While Combination of more classifiers was an effective approach in text classification of defrent languages.

The main objective of this research is to design and implement a new model that utilizes the combination of multi-individual classifiers approach that didn’t use before in Malay sentiment classification with method that integrates SO alongside ML using a lexicon to generate features based on the sentiment words.

This article is organized as the following: Section 2 provides a related work in the field of sentiment analysis (SA) and opinion mining (OM) techniques. In this, section, a short survey on the field of Malay sentiment analysis by ML techniques is presented. Section 3 presents our system architecture (design and implementation). Section 4 presents the results of our system and comparisons with previous work. Section 5 discusses our system results and limitations. Finally, Section 6 presents the conclusion of this paper with final remarks.

## Related work

From previous works, we present an overview regarding the approaches that utilized towards the (SA). First, we survey the (SA) approaches which was used for the English language such as semantic orientation (SO), Machine Learning Approach and Combination Classifier. Second, we discussed previous researches relating to (SA) on the Malay language.

### Semantic orientation (SO)

In the field of (SA), semantic orientation (SO) is an unsupervised learning approach because (SO) does not need preparation of labeled data [16]. Rather, this approach computes how distant a term is towards being negative or positive[17]. By performing unsupervised learning machine, Lexical rules are used in sentiment analysis Rather than basically analysis documents at syntax-level[18]. Kamps, Marx [19] had proposed a model that used lexical relations amid the sentiment analysis task had proposed a model that made utilization of lexical relations amid the sentiment analysis task. In a follow-up study, Qin, Lu [20] outlined a methodology that is utilizations different surveys that are around a similar domain to mine useful printed data. At that point, they formulate semantic comparability measures to recognize a specific sentiment direction. Esuli and Sebastiani [21] proposed a semi-supervised machine learning process, with WordNet in place of a primary vocabulary asset. In their model, a seed set that is imported from WordNet is considered. In their approach, words with the nearby orientation usually have a close label. They implemented a statistical based approach in order to identify the semantic orientation of the seed terms with gloss categorization. In a study conducted by Peng and Shih [22] an unsupervised learning technique for retrieving opinion terms from a document was proposed using parts of speech patterns. In a case of a specific opinion terms are not documented, it was input to a search engine in order to get top total results. After that, with the use of a compiled sentiment lexicon, the sentiments of obscure expressions are computed relying upon the sentiments of close known opinion terms inside the snippets in the retrieved results. Li and Liu [23] designed a k-means clustering model to cluster text blogs toward two categories, which are a positive category and a negative category. The TF-IDF (term frequency—inverse document frequency) weighting is actualized on the content of a text. Next to that, they have implemented a voting majority approach in order to obtain more accurate results. Chaovalit and Zhou [24] had compared between the n-gram machine-learning models against the semantic orientation approach on movie reviews domain. The results show that n-gram machine learning approach outperformed on semantic orientation approach nevertheless require a great amount of computational time in order to train the model. To determine the effects of created an opinion lexicon, Taboada, Brooke [25] found that manually constructing an opinion lexicon is better than constructing a lexicon automatically in order to generate more accurate lexicons. [26] Proposed a sentiment analyses model called SMARTSA which is a lexicon based model. They developed a hybrid lexicon to improves a global lexicon for sentiment analyses based on domain knowledge. [27] had proposed a model that employments the genetic algorithm to generate a sentiment-Lexicon from the training set data. The lexicon-word alongside with its polarities used to implement a manual classification method to obtain the overall polarity test data sentiment. They noted that stop-words may be useful to include in the lexicon.

### Machine learning approach (ML)

The more suitable machine—learning approach to the SA often is a supervised approach in particular, and text classification techniques in general [28]. Like text classification techniques, dealing with the problem of SA as a topic-based text classification problem by machine learning approach [29]. Any text classification algorithm can be applied, for instance, Support Vector Machine (SVM), Naïve Bayes (NB), and K-Nearest Neighbour (KNN). Authors like Pang, Lee [5], Abbasi, Chen [30] proposed a model that employs three of the more used machine learning classifiers in the task of text classification, which was Naïve Bayes (NB), K-Nearest Neighbour, Support Vector Machine (SVM). Then they implement this new model on a data set to categories it into a positive and negative group. Khan, proposed a Semi-supervised framework for sentiment analysis called SWIMS. The developing of a general-purpose sentiment lexicon, Min–Max–Normalized-SentiMI, is implemented with using Senti-WordNet to be weighted features. Then SVM machine learning classifier used in sentiment classification.

### Combination classifier

Researchers have carried out many studies to compare different types of machine learning (ML) classifiers in addition to making use of machine learning techniques for sentiment classification as pointed out earlier. In the last two decades, extensive studies have been done for classifier combination, which has proved successful in enhancing the performance of a wide range of applications [31, 32].

Polikar [33], Zhou [34] reported that a classifier combination can be considered in the machine learning paradigm in that the combined classifier is used to train multiple learners in resolving one task. Ensemble or classifier combination methods attempt to build a set of hypotheses and combine them for use, which is not the case with ordinary machine learning approaches that try to learn one hypothesis based on the training data [35]. Dasarathy and Sheela [36] conducted one of the earliest studies that deal on ensemble learning, where they employed two or more classifiers and discussed the partitioning of the feature space. In 1990, Hansen and Salamon used an ensemble of similarly configured artificial neural networks (ANN) to enhance the generalisation performance of the ANN [37]. Meanwhile, Wang, Zhang [38] showed that the combination of weak classifiers through Boosting, the predecessor of the suite of AdaBoost algorithms, helped generate a strong classifier in Probably Approximately Correct (PAC) sense. Following these seminal works, many studies relating to ensemble learning have been carried, which frequently appear in the literature under many creative names and ideas [33].

By the rise of deep learning, research in artificial intelligence (AI) has gained new vigor and prominence [39]. In [40], it presented sentiment analysis Utterance-Level model with extrinsic evaluations on deep convolutional neural network algorithem to extract textual features. Likewise, to classify the multimodal heterogeneous fused feature vectors applied Multiple Kernel Learning. [41] designed a first deep learning approach to aspect extraction in sentiment analysis. 7-layer deep convolutional neural network applied to tag each word in opinionated sentences as either aspect or non-aspect word. In this research we combined semantic knowledge and machine learning, in which different different approaches can cover for each other’s flaws[42].

A recent study by [43] involved designing a classifying model that uses a combination of several classifiers as sometimes the lack of quality in a particular classifier can be compensated by the quality of another. It does it by the conclusion the right solution provided by a set of three solutions. The number of the selection algorithms among others are majority approach (simple voting), plural (total) voting. In this work, a voting approach is chosen as the combination classification method due to the simplicity of the application and high performance that obtained when a combined method is used in other text classification fields [44, 45].

### Malay sentiment analysis

We could find only a few volumes of research in the literature that applied opinion mining techniques and sentiment analysis over Malay text. There were several techniques involved in our approaches for the Malay language to apply them over Malay text. Some of these approaches implemented traditional ML techniques that were proposed for other languages, while others, despite the rare endeavour, developed new techniques for Malay text.

In Malay SA, Samsudin, Puteh [46] suggested a model which can carry out the task of Malay sentiment analysis. The data used was unstructured and noisy. However, they could not use extra steps, for example lemmatizing, tokenization, stemming and part of speech to solve the problem of the unstructured and noisy data, because these steps require extra tools that were unavailable for the Malay language. They used three ML classifiers SVM, NB, and k-NN. This study was the first to highlight the Malay sentiment classification problem. Samsudin, Hamdan [47] continued their work to improve Malay sentiment analysis. They proposed numerous pre-processing undertakings and a feature selection technique, named FS, which improved the results of Malay opinion mining while using the three classifiers namely Naïve Bayes (NB), Sequential Minimal Optimization (SMO) and k-Nearest Neighbor (KNN). Samsudin, Puteh [48] discussed the use of a feature selection technique FS in opinion mining (OM) using online messages, that were created by the Malaysians. The experiments showed that the technique was better than the traditional ‘filter’ typed feature selection techniques like. Categorical Proportional Difference (CPD), Document Frequency (DF), Information Gain (IG), and CHI Square (CHI). Samsudin, Hamdan [47], Samsudin, Puteh [48], focused on improving the performance of machine learning by using some pre-processing activities and a feature selection. Isa, Puteh [49] considered pre-processing methods for the stemming text in the Malay language using the Reverse Porter Algorithm (RPA) and Backward Forward Algorithm (BFA). After testing, their model’s results showed some enhancement in processing time (when compared to the backward-forward technique), which was an advantage in this model. The performance of the model, during the task of sentiment analysis, was revealed to be similar to using both stemming technique types. Puteh, Isa [50] used an Artificial Immune System (AIS), called Negative Selection Algorithm (NSA), as the individual classifier in sentiment mining for the SAM News Malay newspaper. NSA was able to sentiment mine the newspaper’s data while it was in a standard language. Several problems occurred when major an important detector word when the data did not use a standard language. Additionally, the NSA sentiment-mining model required clean data to operate accurately.

On the contrary, not much of the works were handled using the lexicon approach. According to Chaovalit and Zhou [24], the semantic orientation approach was found to be more efficient but slightly less accurate for use in applications such as Twitter. Furthermore, classifying these data needed no prior training. In general, the semantic orientation approach was found to be practically feasible for automatically mining opinions from unstructured data.

The previous work [15], was more related to our current research. The work dealt with building a hybrid SA model for Malay sentiment analysis. This was achieved by combining both semantic orientation and machine learning techniques through the use of k-NN with a set of features based on the lexicon. Using these features that were based on a polarity lexicon with different classifiers, such as NB, DBN, and SVM, a slight system improvement could be achieved. However, on employing combination classifiers approaches, we could notice significant improvements. The current study is an extension of our earlier research. We used the set of 13 features (as shown in Table 1) that was utilized in [15] to test another traditional classifier for improving the accurate result of our current hybrid model. The main differences compared to our previous work are the use of different machine-learning classifiers and the merging of the hybrid and combination classifiers methods.

**Table 1:** Features extracted for each review.

Feature Set Name	Feature Name	
Sentiment words presence-level features	F1	Presence of positive words
	F2	Presence of negative words.
	F3	Presence of positive words in proportion to the presence of negative words.
	F4	Frequency of positive words in proportion to the frequency of negative words.
Sentence-level features	F5	Cumulative frequency of positive words in the first three sentences.
	F6	Cumulative frequency of negative words in the first three sentences.
	F7	Presence of first positive words synonyms in the first three sentences.
	F8	Presence of first negative words synonyms in the first three sentences.
Sentiment words polarity level features	F9	Weighted probabilities of a positive review
	F10	Weighted probabilities of a negative review.
Subjective words conditional probability features	F11	Average conditional probability of positive subjective words.
	F12	Average conditional probability of negative subjective words.
	F13	Standard deviation of the conditional probability of the subjective words.

In recent years, two studies have attempted on Malay sentiment analysis include [51, 52]. Alfred, Yee [51] proposed a model involved three machines learning classifier NB, KNN, and SVM. They discuss the Issues and parameters that affecting Malay sentiment analysis of news headlines using machine learning approaches. Unlike Alfred, Hasbullah, Maynard [52] reported a semantic Role Labeling (SRL) techniques to filter and classify the public sentiment reviews. The dataset collected from official Malaysian government leaders’ social media sites. Meanwhile, they investigated the effects of public sentiment over Malaysian government officials for policy making and the future development in Malaysia. In addition, a Malay SA and other languages had mentioned as a multilingual sentiment analysis task by Chaturvedi, Cambria [53], [54].

There are many limitations in Malay sentiment classifications researches. Most of the previous research in Malay sentiment classification that conducts the machine learning approach focused on the pre-processing phase [47–49]. This is because of the nature of reviews that were usually written in unstructured language or mix language (i.e. Malay and English) by Malay natives on various online communication applications. Conversely, not many works handled with the lexicon approach. According to [24], the semantic orientation (SO) approach is slightly less accurate but is more efficiency to use in applications such as (Twitter) because no prior training is required in order to classify the data. Overall, the semantic orientation approach almost feasible to mine opinions from unstructured data automatically.

## System description

**Fig 1:**
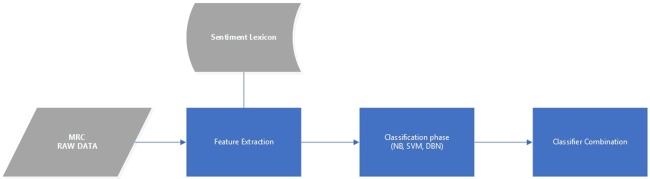
Illustrates the model architecture.

Our recommended solution is the use of a combination-supervised technique, which operates on the document level, to conduct sentiment analysis. The methodology makes use of the raw data (Malay Reviews Corpus) to build a Malay sentiment classification model. First, the partial, noise and incompatible data are removed by pre-processing the raw data. Second, the pre-processed data were then fed through a feature extraction phase. Here, we employed a Malay sentiment lexicon to get values of a pre-defined set of features (sentiment word) from each review. In this model, as shown in Table 1, each review of the raw data was presented in terms of row values for each feature. Third, these values were used as inputs for each of the three machine learning classifiers NB, DBN. and SVM. Fourth, the outputs of the three machine learning classifiers were taken and integrated using the combination method to classify the review as being either negative or positive. (Fig 1) illustrates the architecture of this model.

In this study, we employed a total of 2,478 Malay sentiment-lexicon phrases and words. Each word and phrase were assigned with a synonym and stored, and with the help of more than one Malay native speaker, the polarity was manually allotted a score. We used the English WordNet to gather further accurate sentiment words commonly used in English. These English words were then converted into an equivalent meaning word in the Malay language to categories them as sentiment words in the lexicon. In the Malay sentiment lexicon, each word was associated of each word with its synonyms and allotted a value ranging from 5 (strongly positive) to −5 (strongly negative) by a Malay native speakers. association of each word with its synonym Table 2 shows the association of each word with its synonym.

**Table 2:** Sample of Malay sentiment lexicon.

Word in English	Malay word	Malay synonym	Polarity
Like	Seperti	Bagai	2
Superb	Hebat	Bagus	5
Bad	Buruk	Jahat	-3

### Malay Corpus processing

**Fig 2:**
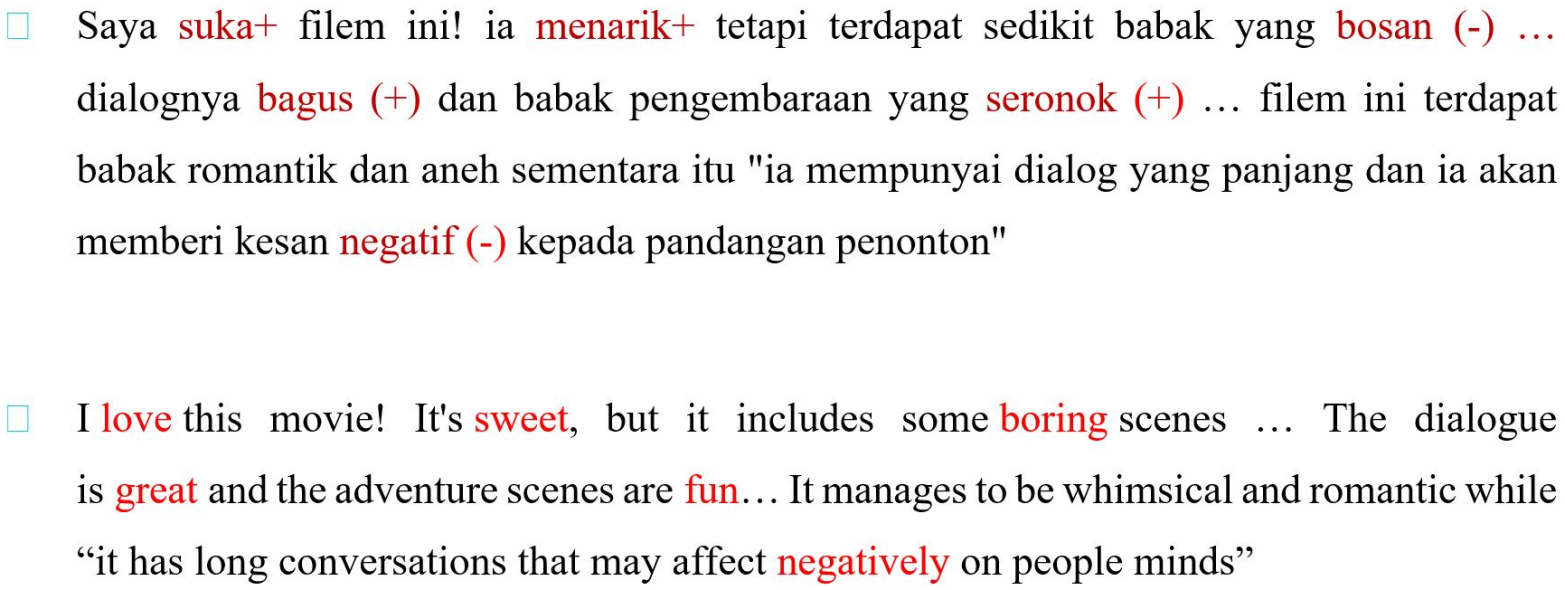
One sample of the reviews.

There were no ready-to-use Malaysian reviews data available on the Web. Therefor the performance measurement of this classification model was in agreement with the Malay Reviews Corpus (MRC) that gathered information from numerous online blogs and forums of Malaysian website. The core websites that contributed to this study were ‘http://www.putera.com’ and ‘http://www.mesra.net’. Many websites were considered to ensure that the collected data would represent online reviews by Malaysian communities. The MRC contained almost 2,000 reviews of which 1,000 were deemed as positive, while the remaining 1,000 were considered negative. (Fig 2) presents sample one of the reviews.

### Pre-processing

The gathered data were raw in nature. Thus, it was imperative to pre-process the raw data in order to apply classifier. The pre-processing task comprises cleaning, tokenisation, normalization and removal of stop words in a text. The input text should be clean from punctuation marks such as commas and periods. Duplicate words may deviate or modify the overall sentiment of the text and must be removed to present as input text for sentiment analysis. The original meaning of the word deviates due to repeated characters in words such as ‘loooonnng’. Thus, such words with repeated characters need to be brought to their original form. Stop words such as a, an, this, the, and which are very common and need to be removed as these do not determine the text sentiment. A list of ready-to-use stop words is available online for the Malay language. Table 3 presents a sample of the Malay stop words with their corresponding English translation.

**Table 3:** Sample of the Malay stop words.

Malay stop words	English translation
Adalah	Be
Itu	It
Selepas	After
Mereka	They

### Feature extraction

In feature extraction methods, we represented each review to a set of 13 features by used the lexicon to tag the existing words in the review along with its polarity, an example is shown in (Fig 2) to clarify that. Then we simply calculated all features mentioned in Table 1, for each review as it mentions previously.

### Naive Bayes (NB)

The Naive Bayes (NB) classifier is commonly used for the review classification. The algorithm can determine the rear possibilities of the classes to relate the review with the help of a feature vector table. The review is then assigned to the class with the maximum rear possibility. Normally employed two models of the Naïve Bayes approach, multinomial and Bernoulli’s multivariate, for text classification. The Naïve Bayes is a stochastic model for generating documents, which follows the Bayes’ rule. The classification of the potential class, c*, for a new document, d, can be calculated by the following Eq (1):

$$c^{*}={max}_{c\;}p(c/d)$$ (1)

The NB classifier is used to compute the posterior probability as follows:

$$p(c_{j}|d_{i})=\frac{p(c_{j})p(c_{j}|d_{i})}{p(d_{i})}$$ (2)

Where ***p*(*c*_*j*_|*d*_*i*_)** represents the later probability of the class *c*_*j*_ with a new document ***d***_***i***_ as the input and ***p*(*c*_*j*_)** as the probability of the class *c*_*j*_, which may be computed by the following equation:

$$P(c_{j})=\frac{N_{i}}{N}$$ (3)

Where *N*_*i*_ represents the total number of the documents classified in the class, *c*_*j*_, and *N* represents the count of documents in all classes. ***P*(*d*|*c*_*j*_)** is the probability of a document *d* when a class *c*_*j*_ is the input and ***P*(*d*)** are the probability of document *d*.

### Support vector machines (SVM)

Support vector machines (SVMs) come under the relatively new class of machine learning techniques. In the machine learning community, SVM is a widely popular method for text classification and one of the most efficient techniques for text classification as established by many studies [55, 56].

Based on the concept of structural risk minimisation, a derivative from the computational learning theory, the SVM separates the training data points into two classes by using a decision surface. Decisions are made based on the support vectors that are the sole selected efficient elements in the training set.

SVM can be a solution for two-class problems that deal with the optimisation of the separating hyperplane between the two data sets. Assuming *X* to be a set of labeled training points (feature vector) (*x*_1_, *y*_1_),…, (*x*_*n*_, *y*_*n*_), and each training point *x*_*i*_ ∈ *RN* is assigned a label *y*_*i*_ ∈ {−1, +1}, where *i* = 1,…, *n*. The goal here is to calculate the function ***f*(*x*) = *w*.*x*_*i*_ + *b*** and to identify a classifier ***y*(*x*) = *sign*(*f*(*x*))** that can be solved through the following convex optimisation:

$${min}_{\;w,b}\sum_{i=1}^{n}\left[1-y_{i}(w.x_{i}+b)\right]+\frac{\lambda }{2}\|w\|$$ (4)

Let λ be the regularisation parameter, where ***x***_***i***_: feature vectors, ***y***_***i***_: ∈{-1,+1}, w: normal vector to hyperplane, and b: offset of the hyperplane.

### Deep belief network (DBN)

**Fig 3:**
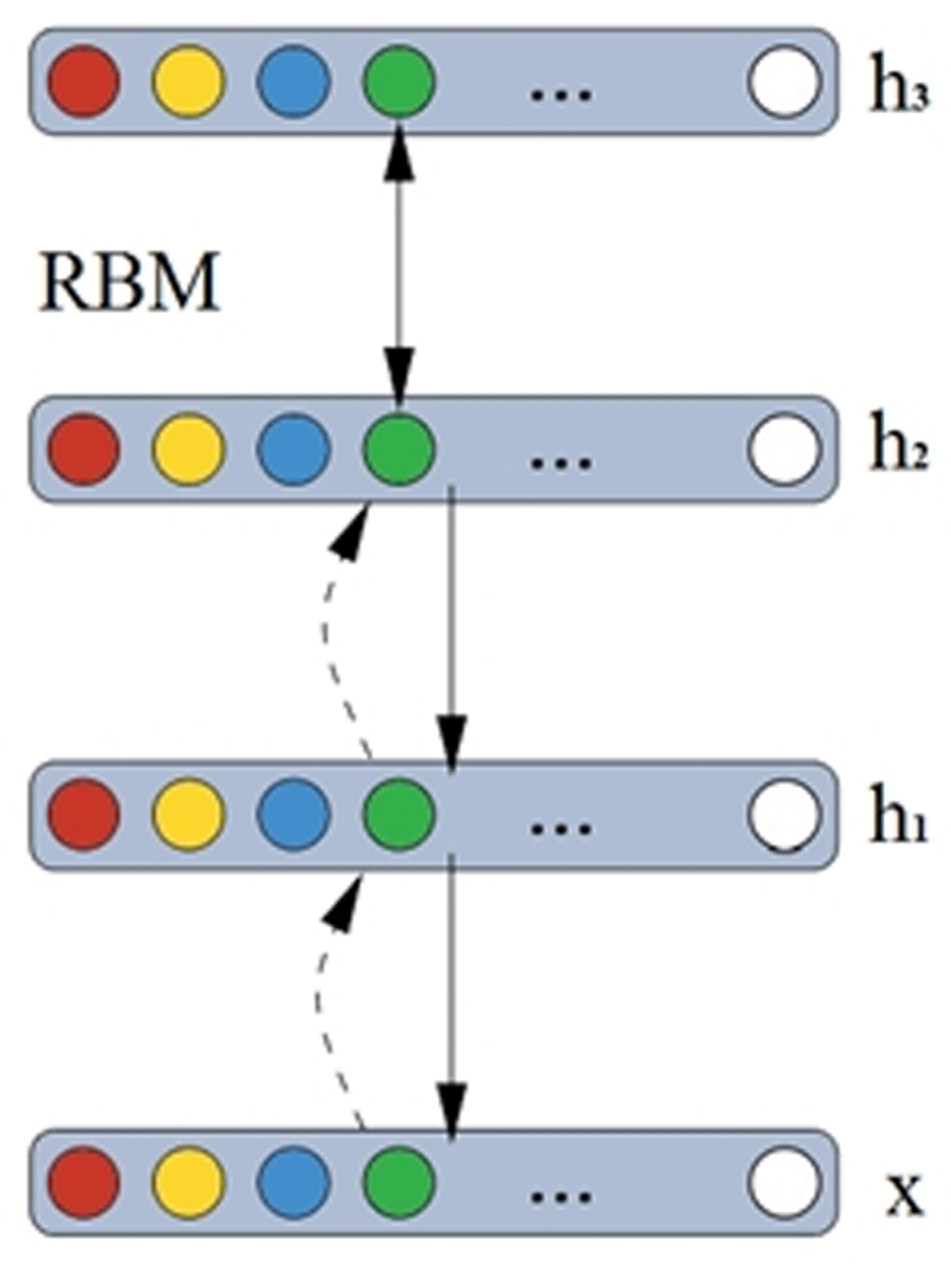
The illustration DBN training processes.

In machine learning, a deep belief network (DBN) is a generative graphical model and is composed of multiple layers of latent variables with connections between the layers but not between units within each layer. In DBN, multiple RBM models are accumulated together and the training process is set from the bottom to the top. However, DBN is different from the multi-layer neural networks. In the multi-layer neural networks, the feature expression performace is robust with the increasing hidden layers. The Backpropagation algorithm may lead to some overfitting problems. In gradient descent method, if the initial value is closer to the optimal solution, the efficient results can be obainted. However, it is difficult to define an optimal initial solution. The DBN can be used to solve this problem efficiently. The training process can be described in Fig 3. The process can be described as the four steps.

1. In the bottom of RBM, the original data is set as training data.
2. The extracted features fromthe bottom of the RBM set as the input training data in the upper layer of RBM.
3. Other higher layer of RBM is repeated by the above two steps.
4. Fine tuning: through these superviseed process, all the parameters are trained supervisedly in the DBN.

In Fig 3, Supposed that an observation is the joint distribution of x and *l* hidden units *h*_*1*_, *h*_*2*_, *…*,*h*_*l*_

The distribution can be expressed by (5):

$$P(x,h^{1},\ldots ,h^{l})=P(x|h^{1})\;.P(h^{1},\ldots ,h^{l})=(\prod_{k=0}^{l-2}P(h^{k}|h^{k+1}))∙P(h^{l-1},h^{l})$$ (5)

where x = *h*^*0*^

In the proposed algorithm, the DBN supervised learning approach is used as the classifer. Above DBN unsupervised learning algorithm, each layer only can ensure that weights of the self layer achieve the optimal solution of the vector mapping. However, the eigenvector mappingis not optimal in the entire DBN. In the DBN supervised learning approach, in the top of the back-propagation networks are used for spreading errors from top to bottom in each layer of RBM to process fine-tuning in the entire DBN networks. The RBM network training model process can be considered as the network weight initialization in BP netwotks, which overcomes the shortcomings of the BP network because the random initialization of the weight parametersis easy to fall into local optimum and long training time.

### Voting classifier combination

The most straightforward voting approach is the majority voting. This technique considers only the most probable class presented by each single classifier to identify the most repeated class label among the output set. In addition, it helps establish whether the overall sentiment of a document is negative or positive. The final classification result is based on the simple majority vote amongst the three classifiers, meaning that two classifiers should agree on the document class out of the three. Weighted majority voting employs a trainable variant of the majority of the voting. In this, every single vote is increased by a weight before the actual voting. The weight for each classifier could be obtained by calculating the accuracies of the classifiers on a validation set. For prediction of an unknown instance, the vote uses each classification model from its sub-process to determine the predicted class to the unknown example with maximum votes.

## Experimental results

Many experiments have been conducted to assess the validity of the proposed methods. First, a number of experiments were established to evaluate the performance of the baseline method. Next, various experiments were conducted for comparing four different feature subsets, namely sentence level, the presence and frequency of sentiment words, sentiment words polarity and subjective words conditional probability. In addition, four classification methods have been by employed for Malay sentiment classification, which are SVMs, Naïve Bayes, DBN, and the combination method. The MRC was used to evaluate the performances of these classification algorithms, and their classification accuracy rate was assessed individually. We conducted a study to evaluate different feature sets and their effects on Malay sentiment analysis. This model allowed efficient integration of different classification algorithms and several feature sets and ensured a more accurate classification procedure.

A 10-fold cross-validation process was employed to evaluate each algorithm. The main idea of this evaluation process is to segment all the dataset into 10 equal size subsamples. In each piece, the number of classes (positive class and negative class) is equal. In our MRC data set (2000 reviews), each piece was (200 reviews) after dividing into 10-fold. The evaluation method input one piece of data set (200 reviews) as test data to the model that was trained with the remaining dataset (1800 reviews). This process was repeated with a different subsample data in each time, and the accuracy of the model was obtained with test data for 10 iteration time. Then the 10-fold results will be averaged to produce a single estimation.

### Baseline model experiments

Initially, SVM, NB and DBN classifiers were applied as a baseline to the entire Unigram feature space. This helped us to assess the overall performance of the classifiers on Malay sentiment analysis without using any features. Table 4 showed the experimental results employing the SVM, NB, and DBN classifiers. Compared with SVM, NB, DBN and classifier combination classifiers performance, the best result was the combination classifier, which was selected as a baseline classifier—in this case, since the classifier combination one achieved the accuracy rate with 80.90%. Furthermore, a comparative evaluation of the sentiment-based features was performed to determine the effectiveness of different feature sets as shown in Table 1. We applied machine-learning classifiers (SVM, NB, DBN and the combination approach) for all features to assess the importance of these features. Furthermore, the impact and the relevance of different sets of features were evaluated for sentiment classification on the MRC.

**Table 4:** Performance of the NB, SVM, and DBN classifiers.

FEATURE TYPE	CLASSIFIER	F-MEASURE%
Unigram	NB	75.20
Unigram	SVM	76.05
Unigram	DBN	80.02
Unigram	Classifier Combination	80.90

### Evaluation of NB, SVM, DBN and combination classifier

**Fig 4:**
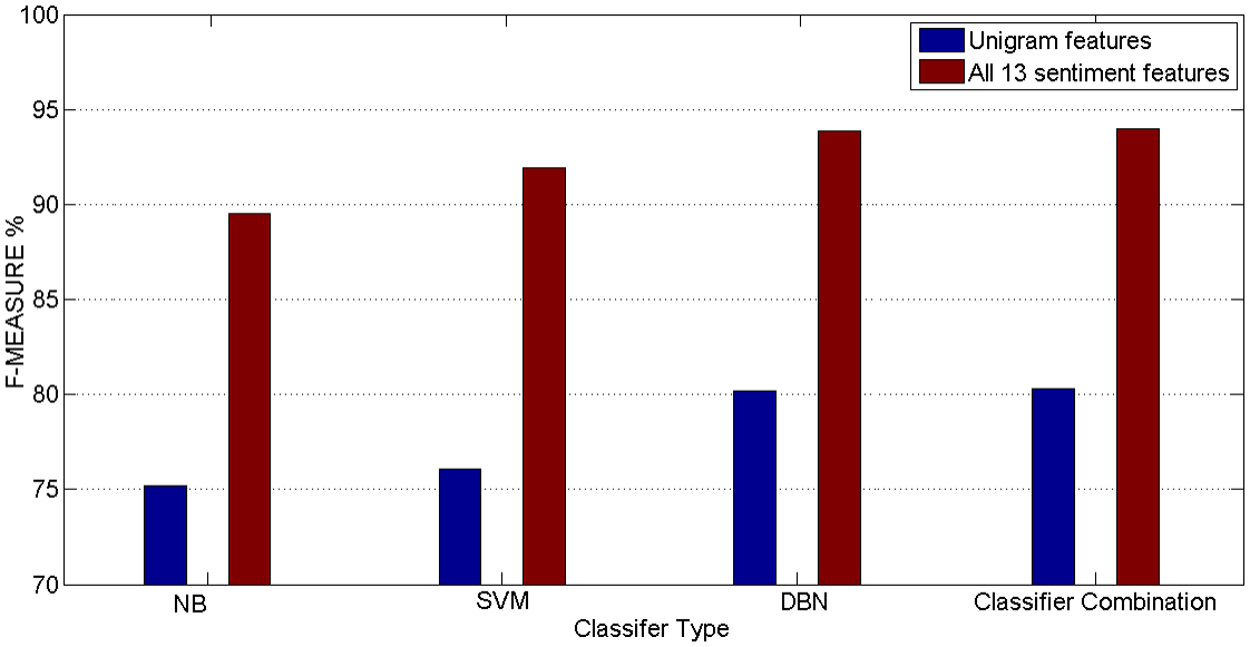
Illustrates highest results of DBN, NB, SVM and classifier combination + 13 sentiment features and Unigram features.

A 10-fold cross-validation procedure was utilised to apply DBN, NB, SVM and combination classifiers to the test set. Table 5 presents the data on the accuracy of the DBN, NB, SVM and combination classifier in terms of F-measure of the Malay sentiment analysis. The selected feature was marked by ‘1’ symbol, and consecutively the obtained accuracy is displayed for the combined selected features. The 54 runs testing were set for evaluating the classifiers accuracy. From run (1) to run (9), the full features for every set were selected for evaluating the classifiers accuracy, which was labelled by Group 1. From run (10) to run (53), the parts features of every set or the full features for every set combined with the parts features of every set were selected for evaluating the classifiers accuracy, which was labelled by Group 2. The No.54 run will be analyzed in Fig 4.

**Table 5:** Measure for NB, SVM, DBN and combination (Comb.) classifiers.

NO	F1	F2	F3	F4	F5	F6	F7	F8	F9	F10	F11	F12	F13	NB	SVM	DBN	Comb.
1.	1	1	1	1										86.29	91.50	91.80	92.44
2.					1	1	1	1						88.81	88.88	90.00	90.82
3.					1	1	1	1	1	1				86.23	88.81	90.20	91.78
4.											1	1	1	84.32	91.55	93.01	93.60
5.	1	1	1	1	1	1	1	1						88.70	90.36	91.45	92.60
6.					1	1	1	1	1	1				87.54	90.16	91.28	92.28
7.									1	1	1	1	1	88.65	90.24	91.20	91.86
8.	1	1	1	1	1	1	1	1	1	1				88.88	92.17	92.24	92.73
9.					1	1	1	1	1	1	1	1	1	88.35	91.52	93.00	93.54
10.	1				1				1		1			86.74	91.22	91.88	92.22
11.		1				1				1		1		85.57	90.94	91.55	92.00
12.			1		1				1				1	86.08	91.10	91.45	92.72
13.				1		1	1	1						86.62	90.31	91.85	92.54
14.	1	1			1	1			1	1	1	1		87.24	90.10	93.08	93.66
15.			1	1	1	1			1	1		1	1	86.89	91.13	92.10	92.76
16.	1		1		1	1		1	1	1	1		1	87.44	91.20	93.26	93.40
17.	1		1	1	1	1	1		1	1	1	1		87.24	91.15	93.20	93.64
18.	1				1									82.66	90.60	92.88	92.94
19.		1						1						82.87	90.16	91.24	92.19
20.			1								1			83.30	90.54	91.90	92.60
21.				1		1								82.87	89.79	91.20	92.16
22.	1							1						82.76	90.84	91.90	92.22
23.		1										1		82.68	90.45	91.42	92.80
24.			1										1	86.48	89.79	91.30	92.16
25.	1				1				1					84.90	90.22	93.56	92.35
26.		1				1		1						85.14	90.22	93.10	93.86
27.			1		1						1			85.63	90.60	91.80	92.55
28.				1					1			1		87.74	90.01	91.40	92.98
29.	1				1								1	87.25	90.38	90.60	92.24
30.	1				1				1		1			84.06	90.38	90.45	92.11
31.		1				1				1		1		84.80	90.03	91.80	92.07
32.			1		1			1					1	87.20	90.11	93.40	93.86
33.				1		1				1		1		85.00	90.33	91.80	92.78
34.	1	1												83.88	90.22	91.54	92.23
35.	1		1											87.75	90.44	93.56	93.88
36.		1	1											83.16	89.24	90.77	92.73
37.			1	1										83.60	90.86	91.22	92.48
38.	1					1								83.60	90.38	91.00	92.60
39.		1						1						84.05	89.93	91.24	92.45
40.			1						1					84.10	90.32	91.25	92.34
41.				1						1				84.34	89.90	90.60	92.15
42.	1										1			83.99	90.71	91.89	92.87
43.		1										1		84.88	90.49	91.32	92.48
44.			1										1	84.05	90.84	91.90	92.82
45.					1		1							81.62	89.98	91.47	92.97
46.	1	1			1									84.92	89.98	91.02	92.80
47.			1			1	1							86.55	90.14	90.78	92.16
48.	1				1				1					85.02	89.69	91.26	92.56
49.		1							1		1			84.80	90.08	93.20	93.77
50.			1							1		1		87.56	89.66	91.90	92.98
51.				1	1							1		84.78	90.48	91.47	92.76
52.							1		1		1			88.53	90.26	91.22	92.84
53.						1				1		1		87.55	90.62	91.18	92.46
54.	1	1	1	1	1	1	1	1	1	1	1	1	1	89.52	92.74	94.10	94.48

In Group 1, for run number (2), (5) and (8), the NB classifier was applied to test a set of sentence-level features (F5, F6, F7, and F8). The best three results were obtained, which were 88.81%, 88.70% and 88.88%. In addition, compared with Tables 4 and 5, the quality of Malay sentiment analysis by the NB classification model was affected by the use of feature sets. The results suggested that the use of the feature sets can only outperform the baseline model trained from unigrams. Based on the average F-measures of the Malay sentiment analysis of run number (8), by combining the feature sets of Sentiment words presence-level features, sentence-level features and sentiment words polarity level features. This combination yielded best result in Group 1 of 92.80%.

In Group 1, the SVM classifier was applied by using feature sets to obtain the results in the third column. Table 5 presented the accuracy of the Malay sentiment analysis in terms of F-measure by applying the SVM classifier to different feature sets. The run number (4) that used only the features (F11, F12, and F13) showed a result of 91.55%. This indicates clearly the positive effect on the performance of the SVM classifier. This result was close to the highest value of the SVM result obtained from all performed experimental results. The highest result of 92.17% in run (8) was obtained employing the sentiment words presence-level features, the sentence-level features and the sentiment words polarity level features. Compared with Tables 4, the used feature sets led to increasing performance of the baseline model. It had a clear impact on the quality of Malay sentiment analysis that employed the SVM classifier. As observed from the results of this experiment and the two previous experiments, the SVM classifier led to better results than those obtained by means of the NB classifier. This revealed that the best individual machine learning technique for Malay sentiment analysis was the SVM classifier. In Group 2, run number (10), (16) and (17) displayed the highest three accuracies. Based on the average F-measures of the Malay sentiment analysis of run number (10), the SVM classifier’s performance is greater than for the NB classifier by combining the feature sets of F1(Presence of positive words) F5 (Cumulative frequency of positive words in the first three sentences), F9 (Weighted probabilities of a positive review) and F11 (Average conditional probability of positive subjective words).

In Group 1, as observed from Table 5 of the DBN classifier, run number (4),(8) and (9) displayed the highest three accuracies. Based on the average F-measures of the Malay sentiment analysis, subjective words conditional probability features (F11, F12 and F13) enhanced the DBN classifier’s performance. The best result obtained 93.01% in run number (4). As noticed from these results and from our previous experiments, the performance of DBN classifier was better than the NB and SVM classifiers. This also meant that the effect of the feature set on the DBN classifier’s performance is greater than for NB and SVM classifiers. In Group 2, run number (25), (32) and (35) displayed the highest three accuracies. Based on the average F-measures of the Malay sentiment analysis of run number (25), the DBN classifier’s performance is greater than for NB and SVM classifier by combining the feature sets of F1 presence of positive words F5 Cumulative frequency of positive words in the first three sentences and F9 Weighted probabilities of a positive review.

In Group 1, in the last column of Table 5, it shown shows the accuracy of the Malay sentiment analysis in terms of the F-measure by employing the combination algorithm with different sets of features. The last column of Table 5 empirically evaluated a classifier combination method. In this combination, three classifiers (SVM, NB and DBN) were integrated together for Malay sentiment analysis in order to test the various features set impacts and indicate impacts of the combination classifier approach. Compared with all results in Table 4, it had a clear impact on the quality of Malay sentiment analysis that employed the combination classifier model. As observed from the results of this experiment and the two all previous experiments, the combination classifier led to the best results among the DBN classifier, the NB classifier and the SVM classifier in the baseline model. The use of the feature sets showed noticeable improvement in Table 5. The combination model with applied feature set always showed the highest results. As seen in the last column of Table 5, run number (4), (6) and (8) showed the highest three results. These results performed the highest value obtained from all performed experimental results among the DBN classifier, the NB classifier and the SVM classifier. In Group 2, run number (14), (26) and (41) displayed the highest three accuracies. Based on the average F-measures of the Malay sentiment analysis, the combination classifier’s performance was the best compared with DBN classifier, the NB classifier and the SVM classifier.

## Discussion

The highest results from classifiers were applied to the entire document-term feature space (Unigram Features) presented in Table 4. Nevertheless, the experimental results employing the SVM, NB, DBN, and Classifier Combination were applied to a Sentiment Features sequentially as well.

First, among the three individual classifiers (Naïve Bayes, Support vector machine, and DBN), the DBN classifier showed the highest result. This led us to select DBN as the baseline classifier for our model in this paper.

Second, the performance of the classifier combination method was greater than other individual classifiers in Malay sentiment analysis. Moreover, the results obtained by employing the classifier combination method were higher when to compare with those obtained by the baseline classifier (DBN). These results led us to infer that the classifier combination method was the most suitable technique for Malay sentiment analysis as we combined the individual strength of each method. When various individual classifiers agree on classifying correctly in most of the cases and disagree on classifying small cases only (when one of them becomes wrong), then combining these classifiers yields higher results. As well, combining the decisions of various single classifiers (several experts) yields higher results and is better than individual classifier (one expert).

Third, as shown in Tables 4 and 5, it is evident that the combination model greatly affacts the implementation of the quality of Malay sentiment analysis. The combination model can achieve the highest results among DBN, NB, SVM and Classifier Combination + 13 sentiment features and Unigram features. Thus, we recommend the implementation of the sentiment features to aid in the task of Malay sentiment analysis models.

In Fig 4, the No.(54) result in Table 5 is used to compare the classifation accuracies. The baseline results from all classifiers + Unigram features are compared with the (SVM, NB DBN, and Classifier Combination) + all 13 sentiment features. Feature extraction improved the performance of Malay sentiment-based classification. Furthermore. Meanwhile, classifier combination method was better than other classifiers in Malay sentiment analysis. Since, an F-measure value of Classifier Combination + sentiment features achieved 94.48, which was reported with the highest results.

## Conclusion

This paper proposes a Malay sentiment analysis classification model for improving classification performances based on the semantic orientation and machine learning approaches. First, a total of 2,478 Malay sentiment-lexicon phrases and words are assigned with a synonym and stored with the help of more than one Malay native speaker, and the polarity is manually allotted a score. In addition, four classification approaches (Naïve Bayes, SVM, DBN and combination method) are used for the evaluation of Malay sentiment classification by using four subsets of features (presence of sentiment words and frequency, sentence level, sentiment words polarity features and subjective words conditional probability features). Finally, it highlights that the Malay sentiment analysis classification model enhances the classification performances with employing the four-classification approach (Naïve Bayes, SVM, DBN and combined-classification approach). Experimental results show that the combination method, which combines various feature sets and classification algorithms, is able to achieve the best result with an F-measure value of 94.48%., and it is the more efficient way to improve classification performances compared with the existing classifiers.

Future work, as a result of this research we have identified the following future directions. First, we plan to improve the data set with increase its size and standardized our lexicon to make it available online for all researcher. Another research direction will focus on the integration of different algorithms for Malay sentiment analysis such as Deep Learning convolutional multiple kernel learning and deep convolutional neural networks.
